# Herlyn-Werner-Wunderlich syndrome presenting with dysmenorrhea: a case report 

**DOI:** 10.1186/s13256-019-2258-6

**Published:** 2019-10-31

**Authors:** Dilruba Sharmen Nishu, Md. Monir Uddin, Khadija Akter, Shameema Akter, Monira Sarmin, Sartaj Begum

**Affiliations:** 1 Department of Gynaecology and Obstetrics, Cumilla Medical College and Hospital (CuMCH), Cumilla, Bangladesh; 2 Department of Radiology and Imaging, Cumilla Medical College and Hospital (CuMCH), Cumilla, Bangladesh; 3 Dhaka Hospital, Nutrition and Clinical Services Division, International Centre for Diarrhoeal Disease Research, Dhaka, Bangladesh

**Keywords:** Herlyn-Werner-Wunderlich syndrome, Obstructed hemivagina, Müllerian anomaly, Vaginoplasty

## Abstract

**Background:**

Herlyn-Werner-Wunderlich syndrome is a rare congenital anomaly characterized by uterus didelphys, obstructed hemivagina, and ipsilateral renal agenesis. The most common presentation is abdominal pain, dysmenorrhea, and abdominal mass secondary to hematocolpos. We present the first case report on Herlyn-Werner-Wunderlich syndrome from Bangladesh.

**Case presentation:**

A 15-year-old Asian girl presented with lower abdominal pain of 3 months’ duration. She had had menarche 3 months earlier and had a regular menstrual cycle with cyclical abdominal pain. Abdominal examination found a tender mass on the right iliac fossa. Further evaluation with ultrasound revealed distended endometrial cavity filled with complex fluid and nonvisualization of the right kidney. Pelvic magnetic resonance imaging showed absent right kidney and two separate endometrial stripes surrounded by endometrium and a muscular layer. The right endometrial cavity and cervix were distended with blood. This magnetic resonance imaging finding is consistent with Herlyn-Werner-Wunderlich syndrome with uterine didelphyis, right-sided hematometra resulting from obstructed hemivagina, and ipsilateral agenesis of the right kidney. The vaginal septum was resected for vaginoplasty. She was discharged 5 days after surgery and came for follow-up after 7 days. Vaginal examination revealed a healthy wound with no adhesion of the vaginal wall. She also informed us that she had started regular menstruation without any pain 30 days after the operation.

**Conclusion:**

An unusual presentation of regular menstruation and nonspecific abdominal pain delays the diagnosis, which can lead to complications such as endometriosis and infertility. Awareness is required; otherwise, misdiagnosis clearly can occur.

## Background

Herlyn-Werner-Wunderlich (HWW) syndrome, a rare variant of paramesonephric (müllerian) duct anomalies, is characterized by the triad of uterus didelphys with obstructed hemivagina and ipsilateral renal agenesis [1]. Obstructed hemivagina and ipsilateral renal anomaly (OHVIRA) syndrome is another name for it [2]. The most common presentation is abdominal pain, dysmenorrhea, and abdominal mass secondary to hematocolpos [3]. In 1922, Purslow first described this syndrome in a young woman who presented with gradually increasing pelvic pain and a pelvic mass with regular menstruation [4].

We describe a case of a young girl with the triad of uterus didelphys, obstructed hemivagina, and right renal agenesis who was diagnosed by pelvic ultrasound, with confirmation by magnetic resonance imaging (MRI), and successfully managed by transvaginal resection of the vaginal septum.

## Case presentation

**Fig. 1:**
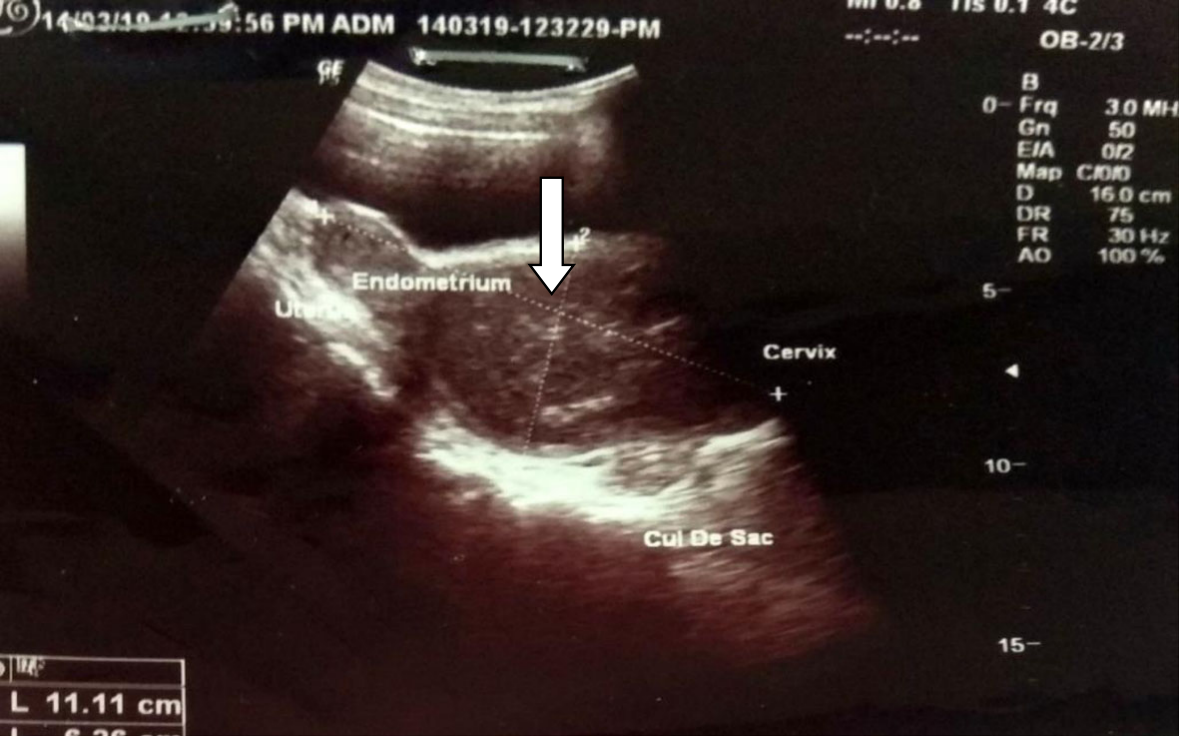
Ultrasonogram of lower abdomen showing endometrial cavity (*arrow*) filled with large, thick collection that extends up to the cervix

**Fig. 2:**
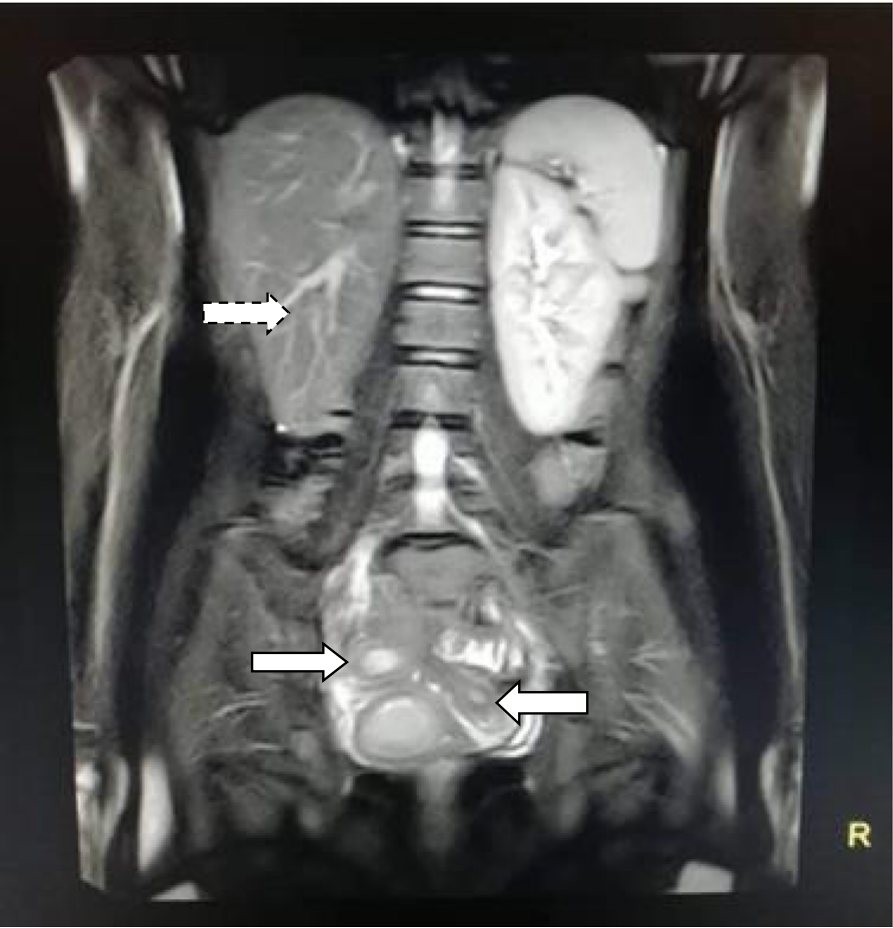
Coronal short tau inversion recovery image showing absence of right kidney (*dashed arrow*) and two separate endometrial stripes (uterine didelphys) surrounded by separate muscle coat (*arrows*)

**Fig. 3:**
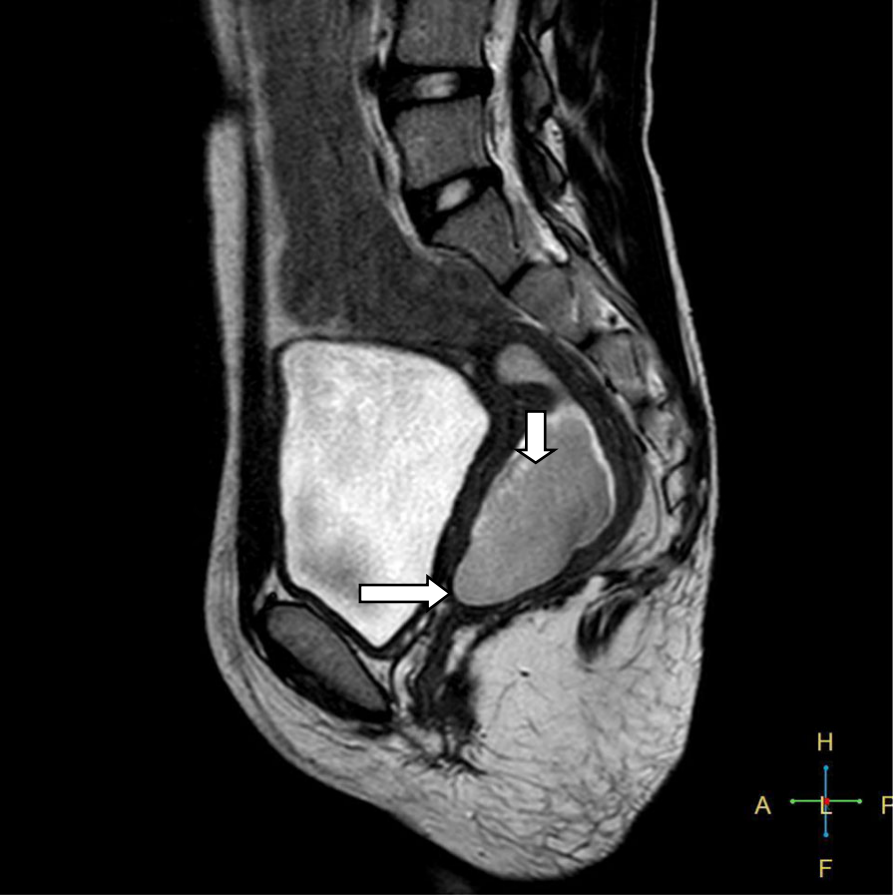
Sagittal T2-weighted image showing dilation of cervical canal (*short arrow*) with abrupt termination at vaginal fornix (*long arrow*), resulting in obstructed hemivagina. Endometrial cavity was mildly dilated and contained hemorrhagic collection

**Fig. 4:**
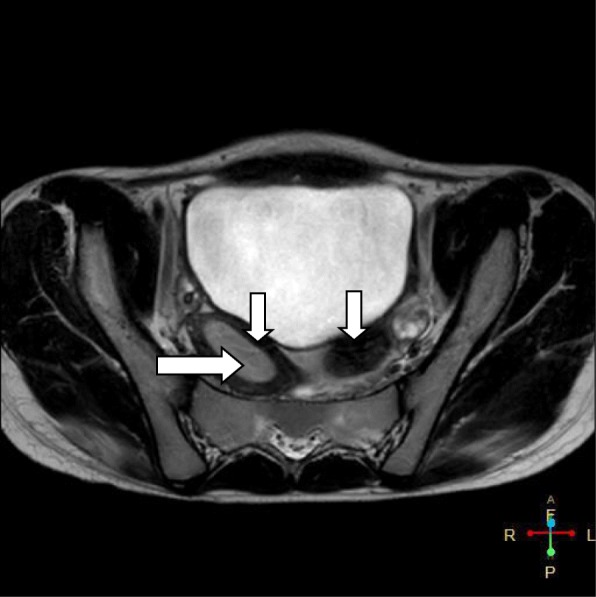
Axial T2-weighted image showing two separate uterine horns (*short arrows*) and distention of right endometrial cavity (*long arrow*)

**Fig. 5:**
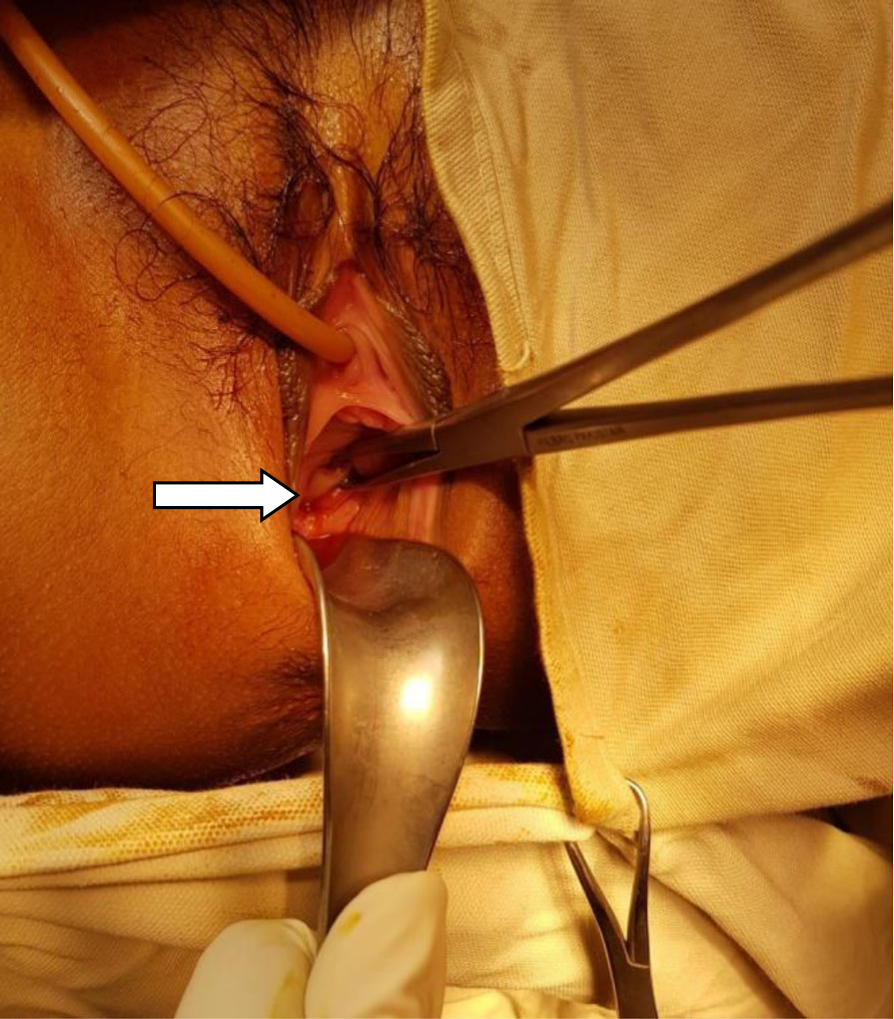
Vaginal septum (*arrow*) was identified perioperatively for resection

**Fig. 6:**
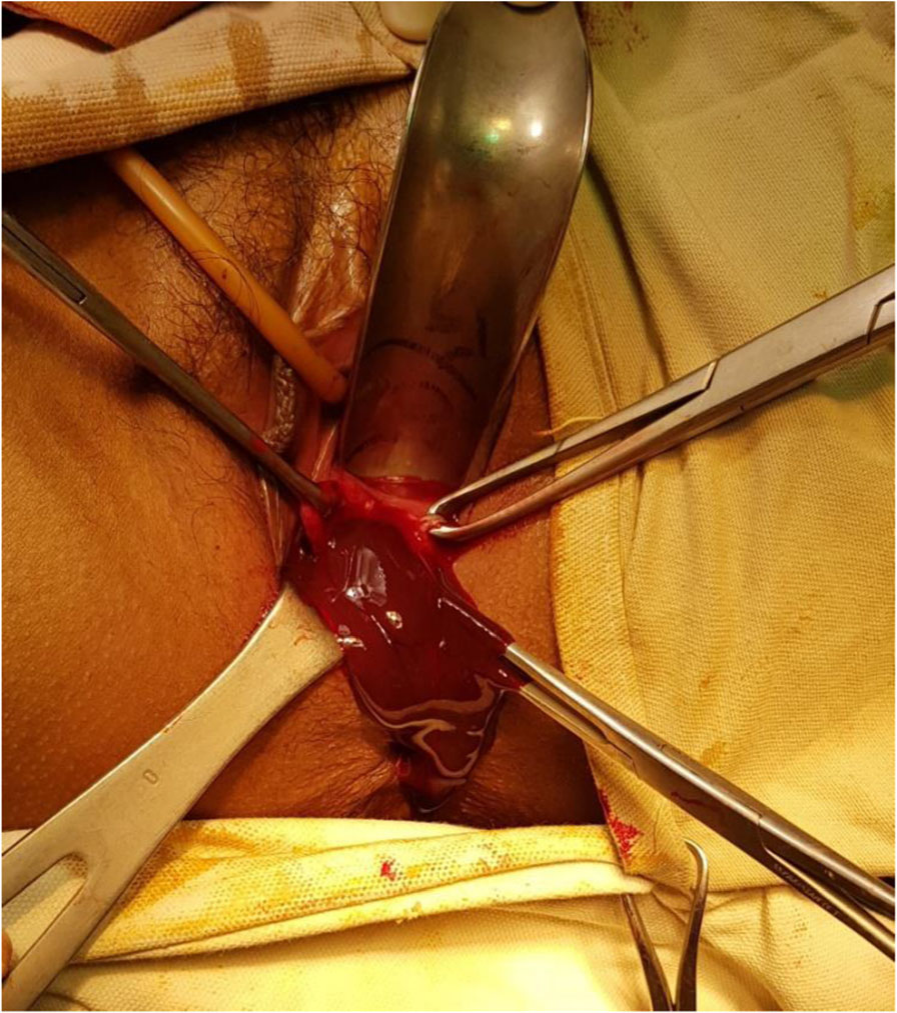
Drainage of tarry inspissated blood

**Fig. 7:**
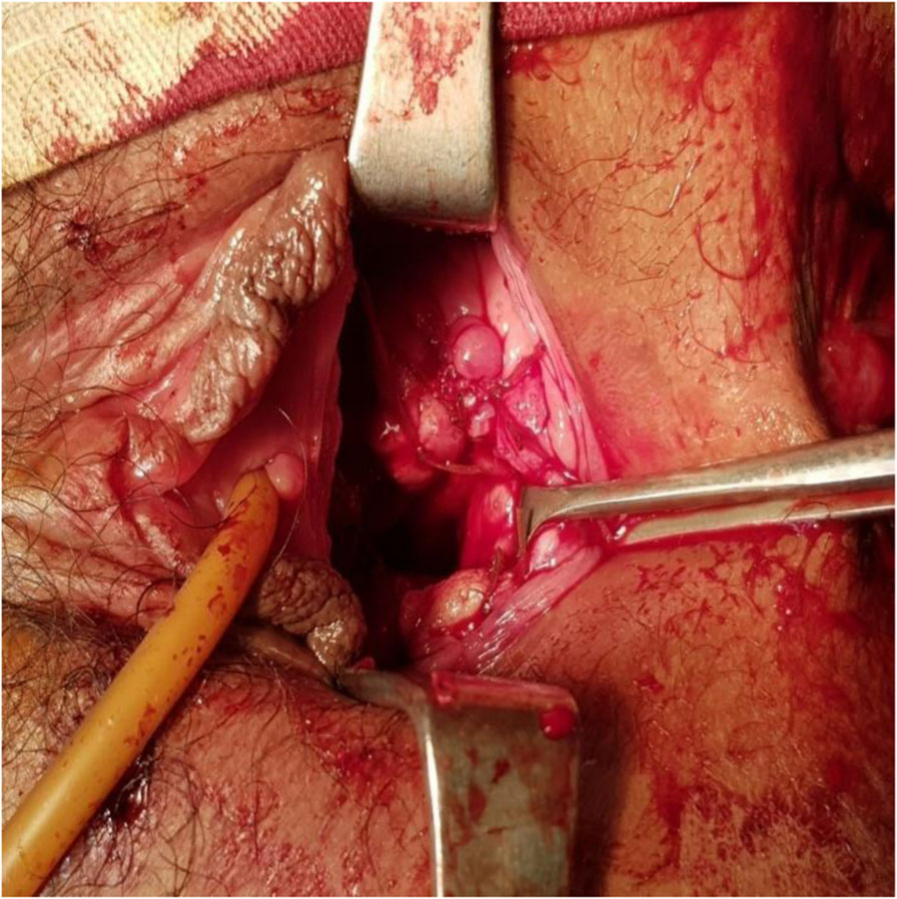
Reconstructed vaginal canal after resection of vaginal septum

A 15-year-old Asian girl presented to the emergency department of Cumilla Medical College and Hospital, Bangladesh, with increasing pain in the right lower abdomen of 3 months’ duration. She experienced severe, colicky pain in the right lower abdomen with the onset of menstruation. Her pain did not radiate and was not associated with fever, vomiting, or urinary complaints. She denied any past medical or surgical history. She had had menarche 3 months earlier and had a regular menstrual cycle with dysmenorrhea and cyclical abdominal pain. For the latter problem, she was prescribed analgesics from a local pharmacy, which resulted in transient improvement of the symptoms. She was born at term of an uncomplicated pregnancy, and she had no family history of congenital diseases. She was not sexually active and did not take contraceptive pills or hormone therapy. She belonged to a middle-class family. Regarding her developmental history, she achieved neck control at 4 months, sitting at 7 months, walking unsteadily from 13 months, and walking steadily from 20 months. Her weight was 33 kg, and her height was 144 cm, both were below the fifth percentile for her age and sex according to the National Center for Health Statistics, Centers for Disease Control and Prevention, and were normal. Her parents were nonconsanguineous. On the day of admission, she was afebrile, and her vital signs were stable except for mild anemia (pulse 84 beats/minute, blood pressure 125/80 mmHg, anemia +, temperature 98 ° F). The results of her other general physical examinations were unremarkable. Abdominal examination found a tender mass on the right iliac fossa. Per rectal examination revealed a mass in the pouch of Douglas. The patient was admitted to the gynecology department, where she was medicated with drugs (analgesic, omeprazole, paracetamol) for relief of symptoms until an MRI scan was obtained and a corrective surgery planned. Routine investigations were done. The patient’s complete blood count was within normal limits with a hemoglobin level of 11.1 g/dl and erythrocyte sedimentation rate of 62 mm/first hour. Her white blood cell count was 12 × 10^9^/L with a differential count of 62.5% neutrophils, 29% lymphocytes, and 6.8% monocytes. Her red blood cell (RBC) count was 3.97 × 10^12^/L. Her platelet count was 431 × 10^9^/L. Routine urine and microscopic examinations showed no features of infection (quantity: sufficient, color: straw, albumin, sugar, and phosphate: nil, pus cells: 4–6/high-power field [HPF], epithelial cells: 3–4/HPF, RBCs: nil). Further evaluation with ultrasound showed distended endometrial cavity filled with complex fluid (Fig. 1) with low-level internal echoes and nonvisualization of the right kidney. A provisional diagnosis of uterus didelphys, hematometra, hematocolpos, and agenesis of the right kidney was made. Pelvic MRI and intravenous urography (IVU) were performed for further evaluation. Pelvic MRI showed two separate endometrial stripes urrounded by endometrium and a muscular layer (Fig. 2). The right endometrial cavity and cervix were distended with blood (Figs. 3 and 4), possibly owing to obstructed right hemivagina. The right kidney was absent (Fig. 2). An MRI finding was suggestive of uterine didelphys with right-sided hematometra resulting from obstructed hemivagina with ipsilateral agenesis of the right kidney (HWW syndrome). IVU revealed an absent or nonexcreting right kidney and normal excreting left kidney. Identification and resection of the vaginal septum were done (Fig. 5) and reached up to the right cervix for the drainage of tarry blood (Fig. 6). Thus vaginal canal was reconstructed (Fig. 7). There were no perioperative or postoperative complications. She was discharged 5 days after surgery.

**Fig. 8:**
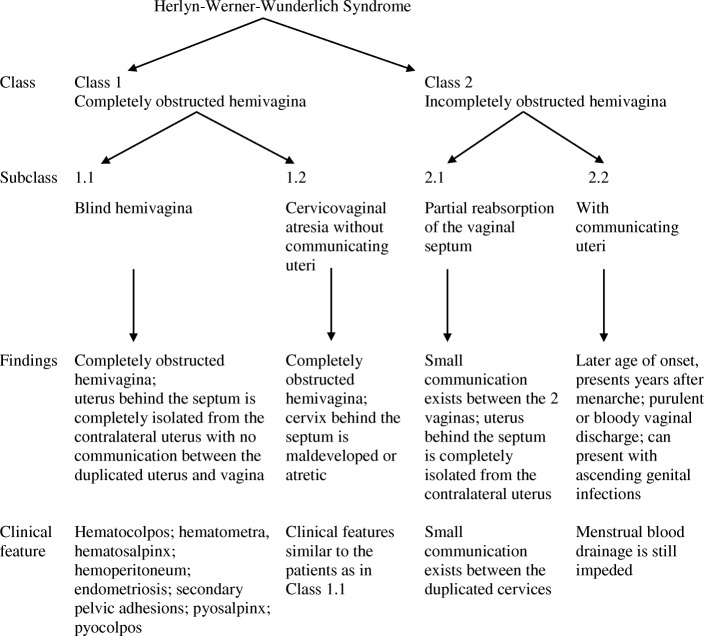
Herlyn-Werner-Wunderlich syndrome based on vaginal morphology [9]

She was seen in regular follow-up. She first came for follow-up 7 days after discharge. She was in good health and had no new complaints. Vaginal examination revealed a healthy wound with no adhesion of the vaginal wall. Thus, her recovery was uneventful. Later, she also informed us that she had started regular menstruation without any pain 30 days after the operation. She visited the hospital for another two follow-up visits almost 1 month apart. Her menstrual cycle was normal, and she had no dysmenorrhea or any other complaints.

## Discussion

We present a case of HWW syndrome in a 15-year-old girl who presented with a regular menstrual cycle and cyclical abdominal pain since her menarche. Ultrasonographic evaluation in a medical college hospital revealed distended endometrial cavity filled with complex fluid and nonvisualization of the right kidney. Pelvic MRI showed an absent right kidney and two separate endometrial stripes surrounded by endometrium and muscular layer. The right endometrial cavity and cervix were distended with blood. Thus, the diagnosis of HWW syndrome was made on the basis of patient history and MRI findings of uterine didelphys, right-sided hematometra resulting from obstructed hemivagina, and ipsilateral agenesis of the right kidney. Thereafter, the vaginal septum was resected for vaginoplasty. The literature revealed that cases can present in a varied way from dysmenorrheal, pelvic, or vaginal mass; abnormal vaginal discharge; acute retention of urine; fever; vomiting to infertility; complicated pregnancy; and labor or endometriosis. This is a case in which the diagnosis was reached early and corrective surgery was done properly and resulted in a better recovery with reestablishment of a regular menstrual cycle without any complications.

HWW syndrome is a triad of obstructed hemivagina, uterine didelphys, and ipsilateral renal agenesis. The true incidence is variable between 0.1% and 3% [2]. The etiology is not well established. Embryological development is influenced by genetic and environmental factors. In HWW syndrome, there is an insult to the paramesonephric system and metanephros [5]. The uterus, fallopian tube, cervix, and upper two-thirds of the vagina develop from the paired paramesonephric ducts. The duct arises from the urogenital ridge [6]. Then, caudally, it runs lateral to the mesonephric duct, and finally, in the midline, it comes in close contact with the paramesonephric duct from the opposite side and fuses to form the uterus, the cervix, and the upper part of the vagina [6]. When they fail to fuse, they produce two hemiuteri and hemicervices, resulting in müllerian anomalies associated with OHVIRA syndrome [7]. An insult to the metanephric diverticulum results in ipsilateral agenesis of the ureter and kidney [8]. Based on the morphology of the vagina, HWW syndrome has been classified as class 1 (completely obstructed hemivagina) and class 2 (incompletely obstructed hemivagina). Both classes have two subclasses. Our patient’s case represents subclass 1.1 with uterus didelphys and blind hemivagina (Fig. 8) [9].

Usually, HWW syndrome remains undiagnosed and asymptomatic during early childhood with normal external genitalia [10]. Classically, a patient with HWW syndrome can present with severe dysmenorrhea a few months to 1 year after attaining menarche. This is what happened in our patient. For her dysmenorrhea, she received symptomatic management. Other patients can present with a pelvic or vaginal mass, abnormal vaginal discharge, acute retention of urine, fever, vomiting [11], infertility, complicated pregnancy and labor [12], or endometriosis. The mean age of presentation is about 15 years [13], which is quite similar to our patient’s case.

For diagnosis, ultrasound and computed tomography are useful. For imaging soft-tissue anatomy and identifying congenital anomalies, MRI is increasingly used. In our patient, the MRI findings of uterine didelphys with right-sided hematometra and obstructed hemivagina with ipsilateral agenesis of the right kidney made the diagnosis easier. Resection of the vaginal septum is the treatment of choice of obstructed hemivagina [1]. We have also followed the same principle. Vaginoplasty was done to reconstruct the vaginal canal with the drainage of tarry blood.

## Conclusion

An unusual presentation of regular menstruation and nonspecific abdominal pain makes the diagnosis of HWW syndrome difficult and requires special clinical suspicion. Early identification warrants awareness of such an anomaly. Ultrasonographic and MRI findings can collectively help to diagnose this rare abnormality. A multidisciplinary approach guided by a gynecologist, radiologist, pediatric specialist, and pediatric surgeon is fundamental to avoid complications and achieve a better outcome.

## Data Availability

We will not be able to share medical imaging data, because they are not fully anonymous.

## Abbreviations

HPF: High-power field
HWW: Herlyn-Werner-Wunderlich
IVU: Intravenous urography
MRI: Magnetic resonance imaging
OHVIRA: Obstructed hemivagina and ipsilateral renal anomaly
RBC: Red blood cell

## References

[1] Aveiro AC, Miranda V, Cabral AJ, Nunes S, Paulo F, Freitas C. Herlyn-Werner-Wunderlich syndrome: a rare cause of pelvic pain in adolescent girls. BMJ Case Rep. 2011;2011:bcr0420114147. 10.1136/bcr.04.2011.4147 PMC3139160 22689557

[2] Smith NA, Laufer MR. Obstructed hemivagina and ipsilateral renal anomaly (OHVIRA) syndrome: management and follow-up. Fertil Steril. 2007;87(4):918–22. 10.1016/j.fertnstert.2006.11.015 17320871

[3] Piccinini PS, Doski J. Herlyn-Werner-Wunderlich syndrome: a case report. Rev Bras Ginecol Obstet. 2015;37(4):192–6. 10.1590/SO100-720320150005077 25992503

[4] Purslow C. A case of unilateral hæmatokolpos, hæmatometra and hæmatosalpinx. BJOG Int J Obstet Gynaecol. 1922;29(4):643.

[5] Kimble RM, Kimble RM. The obstructed hemivagina, ipsilateral renal anomaly, uterus didelphys triad. Fertil Steril. 2010;93(4):e15. 10.1016/j.fertnstert.2009.08.046 20117779

[6] Sadler TW. Langman’s medical embryology. 12th ed. Philadelphia: Lippincott Williams & Wilkins; 2012.

[7] Acién P, Acien MI. The history of female genital tract malformation classifications and proposal of an updated system. Hum Reprod Update. 2011;17(5):693–705. 10.1093/humupd/dmr021 21727142

[8] El-Gohary MA. Uterus didelphys with obstructed hemivagina and ipsilateral renal anomaly (OHVIRA syndrome): a case report. J Pediatr Surg Case Rep. 2014;2(9):410–2.

[9] Zhu L, Chen N, Tong JL, Wang W, Zhang L, Lang JH. New classification of Herlyn-Werner-Wunderlich syndrome. Chin Med J. 2015;128(2):222. 10.4103/0366-6999.149208 PMC4837842 25591566

[10] Gupta N, Gandhi D, Gupta S, Goyal P, Li S, Kumar Y. A variant of Herlyn-Werner-Wunderlich syndrome presenting with acute abdomen: a case report and review of literature. Glob Pediatr Health. 2018;5:2333794X18769817. 10.1177/2333794X18769817 PMC5946351 29761137

[11] Mandava A, Prabhakar R, Smitha S. OHVIRA syndrome (obstructed hemivagina and ipsilateral renal anomaly) with uterus didelphys, an unusual presentation. J Pediatr Adolesc Gynecol. 2012;25(2):e23–e5. 10.1016/j.jpag.2011.11.004 22421561

[12] Shavell VI, Montgomery SE, Johnson SC, Diamond MP, Berman JM. Complete septate uterus, obstructed hemivagina, and ipsilateral renal anomaly: pregnancy course complicated by a rare urogenital anomaly. Arch Gynecol Obstet. 2009;280(3):449–52. 10.1007/s00404-008-0919-6 19132379

[13] Bajaj SK, Misra R, Thukral BB, Gupta R. OHVIRA: uterus didelphys, blind hemivagina and ipsilateral renal agenesis: advantage MRI. J Hum Reprod Sci. 2012;5(1):67. 10.4103/0974-1208.97811 PMC3409925 22870020

